# The Concepts of Work and Decent Work in Relationship With Self-Efficacy and Career Adaptability: Research With Quantitative and Qualitative Methods in Adolescence

**DOI:** 10.3389/fpsyg.2021.660721

**Published:** 2021-03-30

**Authors:** Andrea Zammitti, Paola Magnano, Giuseppe Santisi

**Affiliations:** ^1^Department of Educational Sciences, University of Catania, Catania, Italy; ^2^Faculty of Human and Social Sciences, Kore University, Cittadella Universitaria, Enna, Italy

**Keywords:** the meaning of work, the meaning of decent work, self-efficacy, career adaptability, career counseling

## Abstract

The way people make career choices is often influenced by their idea of work. Alongside this concept, there is the idea of decent work, which takes the form of the opportunity, for men and women, to have productive, equal, safe, and rights-based work. We have conducted a study on these two concepts with a group of Italian adolescents, using both quantitative and qualitative methods. We found that most of the participants consider work as a means to obtain economic benefits and satisfy certain values, and decent work as characterized by the respect for rights and duties and economic benefits; a part of the participants fails in giving a definition of decent work or gives a negative definition of it. We deepened the study through quantitative analyses that revealed that those who have a more complex view of work and decent work also have higher levels of self-efficacy and career adaptability. These findings are discussed in the light of previous research and from a perspective that intends to give a contribution to career counseling practices.

## Introduction

The adolescents' conception of work and decent work is an important aspect of career education and vocational guidance process. The times we are living are characterized by rapid changes especially in working places and conditions; the way individuals face these unpredictable situations is affected by the meaning they give to work, including the new perspective of decent work (Di Fabio and Blustein, 2016). While previous research has largely deepened how adolescents interpret work, little research has been done in the field of decent work with this age group.

There are different definitions of the concept of work: Drenth (1991) defined work as a crucial activity for human survival, which allows cultural development, professional fulfillment, and the satisfaction of the needs of life; Richardson (1993) has pointed out that work serves to achieve personal success and satisfaction and also allows to connect the individual to society; Warr (1984) states that work has not only advantages but also disadvantages: the author observed the cost of work in terms of psychological and physical stress and strain. According to Blustein (2006), work is about making people feel like a part of the society they live in.

One of the first studies that addressed the concept of work involved eight different countries (United States, Germany, Israel, Yugoslavia, Japan, Belgium, Holland, and England; Meaning of Working International Research Team, 1987) and identified four main aspects in defining the work: practical aspects of the job, such as wages or results, social aspects related to the possibility of feeling part of society, aspects related to the sense of duty, and aspects related to the stress and commitment associated with work. However, this study involved an adult population, while in recent years the focus has also shifted to young people. For example, Blustein et al. (2002) observed that young adults with a high socioeconomic status viewed work as a means of self-expression, whereas those from a lower socioeconomic background viewed it as a way to earn a living. Furthermore, Chaves et al. (2004) highlighted how culture can influence the way students describe work; these authors noted that urban teenagers describe work as a means of earning money or surviving or as a means of self-expression. Phillips et al. (2002) conversely showed that work can generate anxiety and tension in adolescents; moreover, difficulties in the workplace can be linked to depressive states as adults (Faraci and Tirrito, 2013). Sinisalo (2004) conducted a study in three different periods with Finnish adolescents aged 15 and 16 years. He showed that the representation of work can change over time, and there are gender differences: boys attach greater importance to extrinsic benefits, whereas girls give more importance to intrinsic values.

The non-definitive results found in the previous studies suggest that further research is needed.

Alongside the concept of work, in 1999 the International Labour Organization [International Labour Organization (ILO), 1999] introduced the concept of decent work; it is configured as an opportunity that is given to men and women to obtain productive work in conditions of freedom, equity, safety and respect for human rights, prospects for individual development, and social integration. A job, to be decent, should provide a fair income, job security, protection for workers and their families, opportunities for personal development, social integration, the ability to express concerns and organize into groups, equal opportunities, and equal treatment for all.

In a transdisciplinary study on the concept of decent work, Di Fabio and Maree (2016) distinguished different perspectives: philosophical, legal, economic, sociological, and psychological. The philosophical perspective (Peruzzi, 2015) focuses on the concept of dignity and how it can be influenced by the culture of belonging. The legal perspective (Faioli, 2009) focuses on the labor law system, which should make it easier to access new job opportunities and strengthen employability policies. The economic perspective (United Nations Economic Social Council, 2006) underlines “the right of everyone to the enjoyment of just and favorable conditions of work” (article 7 of the International Covenant on Economic, Social and Cultural Rights), reiterating the concepts of equity, safety, equal opportunities, and respect for rights. The sociological perspective refers to how the concept of decent work has changed over time: the definition given in 1999 by the International Labour Organization was enriched. In 2015, the International Labour Organization has defined decent work as “opportunities for work that is productive (to satisfy individual basic needs and to give a contribution to society) and delivers a fair income, security in the workplace and social protection for families, better prospects for personal development and social integration, freedom for people to express their concerns, organize and participate in the decisions that affect their lives and equality of opportunity and treatment for all women and men” [International Labour Organization (ILO), 2015]. So, the four initial points highlighted by the International Labour Organization (workers' rights, employment, social protection, and social dialogue) evolved into the promotion of employment, the guarantee of labor rights, social protection, which comprises inclusion and promotion of social dialogue. Finally, the psychological perspective, with Burchell et al. (2014), reflects on decent work in terms of (1) job satisfaction, which refers to the expectations of the worker at work and the perception of the quality of work (Agassi, 1982); (2) intrinsic quality of work, which refers to the well-being of workers, also guaranteed by the strategies adopted by employers; and (3) the desirability of work, i.e., the objective characteristics of work that can promote workers' assessment of their situation and contribution (Burchell et al., 2014).

The psychology of career counseling also promotes decent work. The point of view of career counseling psychology focuses on the elements that during the career-building process can contribute to the creation of a meaningful relationship between individual resources and opportunities offered by the environment (Di Nuovo et al., in press). Athanasou (2010) emphasizes the link between decent work and career and promotes the duty of professional counselors to promote equity in professional settings. The International Association for Educational and Vocational Guidance (IAEVG) stresses that “effective educational and vocational guidance and counseling can assist individuals to understand their talents and potential and enable them to plan the appropriate steps to develop essential skills that will lead to personal, educational, economic and social advancement for the individual, family, community, and nation” [The International Association for Educational Vocational Guidance (IAEVG), 2001].

Therefore, the literature review confirms the challenge of promoting research in the area of decent work (Guichard, 2013). Although the research in the last 20 years has focused on the concept of work, the perception of decent work is still poorly understood. A qualitative study on the representation of decent work was conducted by Magnano et al. (2021b), involving adults in conditions of social vulnerability; the results showed that the meaning attributed to the participants to decent work was not very different from the definition of work in general. In the recent literature, there are no qualitative studies that have investigated the representation of decent work in adolescence.

Ferrari et al. (2009) showed that the complexity of the concept of work is linked to different levels of achievement, self-evaluation abilities, self-regulation abilities, and abilities to gather information useful to making a choice. In particular, students with a more complex idea of work presented higher levels in these dimensions. Furthermore, those who describe the work with reference mainly to economic advantages show higher scores for avoidance and lower scores for abilities of planning and self-monitoring, self-regulation abilities, and internality than those who describe the work by referring mainly to psychological advantages.

In the study presented, we first aimed to explore the representation of work and decent work in a sample of Italian adolescents, following a qualitative methodology. Second, following a mixed methodology, we have categorized the representations of the concepts of work, and decent work emerged and verified whether these categories correspond to significant differences in terms of psychological resources, such as self-efficacy and career adaptability. We hypothesized that a more complex representation of the concept of work or decent work may correspond to higher levels of self-efficacy or greater ability to manage the complexity of today's world of work. The rationale of this study has already been underlined in other studies (Ferrari et al., 2009) and refers to the fact that those who can think complexly about work will likely show a greater level of flexibility in managing professional challenges and tasks. However, the innovative element of our research is the inclusion of the concept of decent work and the involvement of a group of adolescents.

The purpose of this article is therefore to fill a gap in the literature: we aim to contribute to enriching knowledge in the field of vocational guidance, providing some suggestions to professionals who are involved in developing career education and career counseling interventions.

## Methods

### Participants

The participants to the study are 308 Italian students attending the second and third grade of middle school. Regarding the gender distribution, the participants were 153 males and 155 females, aged between 11 and 14 years (mean = 12.62, SD = 0.62).

To participate in the research, the students received an invitation from the schools that collaborated with the university in conducting this study. As they are minors, parents were asked to sign their consent to participation and data processing. Data collection took place during school hours, in small groups, and under the supervision of a psychologist expert in vocational guidance, which is a component of the research group. Only the researcher was present during the administration of the survey, and the participants were guaranteed anonymity. The university ethics committee previously approved the research design. The participants gave their informed consent for inclusion before they participated in the study. The study was conducted in accordance with the Declaration of Helsinki, and the protocol was approved by the IERB (Internal Ethics Review Board) of Department of Educational Sciences, University of Catania, November 25, 2020.

### Measures

The research protocol contained the following sections.

#### Biographical Data

Participants were asked to indicate their gender and age.

#### Measurement of Self-Efficacy

To evaluate self-efficacy, General Self-efficacy Scale (Schwarzer, 1993; Di Nuovo and Magnano, 2013) was used. The scale is one-dimensional and composed of 10 items on a five-point Likert scale (from 1 = totally disagree, to 5 = totally agree). Sample item is “indicate how much corresponds to yourself. whatever happens to me, I'm usually able to celebrate.” In this study, Cronbach's α value was 0.79.

#### Measurement of Career Adaptability

Career Adaptability Inventory (Savickas et al.; Italian adaptation by Soresi et al., 2012) was used to measure the four dimensions of career adaptability: control, concern, curiosity, and confidence. The participants were asked to indicate how much they believe to possesses certain abilities on a five-point Likert scale (from 1 = I possess very little this ability, to 5 = I possess this ability very much). Sample items are “reflect on what my future will be like” (measure of control), “decide autonomously” (measure of concern), “explore my living environment” (measure of curiosity), and “overcome obstacles” (measure of confidence). In this study, Cronbach α values for control, concern, curiosity, and confidence were 0.68, 0.67, 0.75, and 0.79, respectively, and 0.89 for the total score.

#### Concepts of Work and Decent Work

To investigate the representation of work and decent work, as suggested by other studies (Ferrari et al., 2009; Zammitti et al., 2020; Magnano et al., 2021b), we used two open questions: what is your definition of work? What is your definition of decent work?

### Procedure

The participants took part voluntarily to the research. The study was conducted in several schools in Catania (Italy). The data were collected at the beginning of the 2019/2020 school year. The students received an invitation to participate to the research, and their parents were asked to sign the informed consent necessary for data processing and administration of the research protocol. The data were collected during school hours, in agreement with their teachers, by a psychologist expert in vocational guidance activities with adolescents, and the participants were guaranteed anonymity. Only the psychologist was present during the data collection in the classroom. Once the data were collected, qualitative analyses were conducted (phase 1). Subsequently, the study was extended with quantitative analyses (phase 2).

In phase 1, to understand how adolescents represent the concepts of work and decent work, we conducted a content analysis related to relevant categories and themes (Hsieh and Shannon, 2005). This type of analysis allows using an inductive approach, based on data, and drawing conclusions without having any starting hypotheses (Hsieh and Shannon, 2005). This procedure is consistent with the Grounded Theory Methods (GTM). GTM allows exploring a domain without an organizational theory (Glaser and Strauss, 1967), making the theory emerge from the data. These practices provide intellectual rigor to organize an investigation (Gasson, 2004; Charmaz, 2006, 2009; Corbin and Strauss, 2008). Much research in GTM is qualitative, but the same rigor can be applied to exploratory studies that use quantitative or mixed—qualitative and quantitative—data (Bryant and Charmaz, 2007; Morse et al., 2009). For this reason, in study 1, when we analyzed the concepts of work and decent work, we did not take into account the definitions given in the introduction, but we preferred that the themes emerged from the data, through an exploratory and therefore mainly qualitative approach. NVivo 12.0 software was used for this type of analysis; first, we subjected the data to a pretreatment; that is, after having transcribed the data in the software, we corrected grammatical errors or words written in dialect. Everything has been reported in Italian. The data analysis with the NVivo software allowed us to identify the most used words and the most used nodes in the description of the concepts. Using a procedure already adopted in other studies (Zammitti et al., 2020), the analyses were conducted separately by two researchers, belonging to the research group. The two researchers met repeatedly and reached an agreement on the coding also through a third researcher (part of the research group). Eight nodes and 488 encodings were identified for the concept of work and 10 nodes and 386 references for the concept of decent work, illustrated in phase 1.

At this point, the judges first considered only the definition of the work and codified the qualitative data to distinguish three groups of students: those who have a complex perception of work, those who possess a moderately complex perception of the concept of work, and those who have a little complex perception. A fourth group composed of those who did not have a clear idea of what work was considered.

For the concept of decent work, it was possible to identify three groups: those who had no idea of decent work or who defined it negatively, those who have a complex perception of the concept of decent work, and those who have a not very complex perception of the concept of decent work.

This allowed us to calculate the differences between the groups regarding career adaptability and self-efficacy, whose results are presented in phase 2.

## Results

### Phase 1: The Concepts of Work and Decent Work

First, we used the “word frequency” query separately for the concepts of work and decent work; this helped us to identify each node (QSR International, 2014). We used the option that allows searching for words that consist of at least five letters and that were present more than four times. In this coding, we have excluded all the articles and adverbs that have been included in the “list of nonsignificant words,” and we have combined the same words expressed in the masculine and feminine or the singular and plural. Table 1 shows the results of this query.

**Table 1:** Word frequency for the concept of work.

**Word**	**Count**	**Word**	**Count**
Work	97	Knowledge	9
Family	62	Passion	8
Earn a living	49	To grow up	8
To be useful	43	Survive	7
Money	42	Learn	7
To gain	41	Rights	6
To live	34	Duty	6
Pleasant	28	Responsibilities	6
Opportunity	22	Satisfaction	5
To go forward	28	Time	5
Future	21	Fatigue	4
Commitment	26	Hobby	4
Activity	20	Sacrifices	4
Capacity	17	Dreams	4
Important	13	Study	4
Society	20	Useful	4
Helping	21	World	4

Based on the word frequency, we have codified different nodes and subcategories, whose frequencies and examples are shown in Table 2. The nodes were (1) economic aspect of work: refers to the perception of work as something that is used to earn money. Examples are “work is an activity that allows you to earn” (participant no. 4) or “work allows you to bring bread home and not starve” (participant no. 116); (2) the satisfaction of values: this node has to do with the idea that work can contribute to the satisfaction of certain values and has been divided into subcategories: (2.1) creating a family, refers to the possibility of creating and maintaining a family, thanks to work. Examples of this node are “work is important to keep the family” (participant no. 135) or “work is an activity that serves exclusively to keep the family going” (participant no. 140); (2.2) personal fulfillment, refers to achieving one's well-being through work. Examples of this node are “work is something that allows you to realize yourself” (participant no. 50) or “work is something that in the future will allow us to feel satisfied and to realize our dreams” (participant no. 152); (2.3) well-being of society, i.e., the fact that work allows people to make their contribution to social well-being. Examples of this node are “work is the contribution that every citizen must give to the State” (participant no. 35) or “work is a task that each individual carries out to help the community in which he/she lives” (participant no. 120). (3) Increasing one's skills. This node refers to the perception of work as something that allows to improve individuals' skills, and examples of this node are “work allows us to learn” (participant no. 45) or “work is a way to demonstrate one's capacity and increase them” (participant no. 117). (4) Preparing for the future. This node refers to the perception of work as something that is needed to prepare for the future. Examples of this node are “work is something for the future” (participant no. 24) or “work helps you prepare for the future” (participant no. 279). (5) Commitment refers to the fact that work involves personal commitment, sacrifice, and a sense of responsibility. Examples of this node are “work is committing oneself” (participant no. 29) or “work is a commitment to always respect to live better” (participant no. 94). We also identified a sixth node that we called “other,” in which we included the answers that did not fit into the previous nodes as they cannot be classified or the non-answers. Examples of this node are “work is something that man does” (participant no. 141) or “work is the profession” (participant no. 153). Table 2 resumed the results of this query.

**Table 2:** Nodes, references, and coverage for the concept of work.

**Node**	**References**	**Coverage**
Economic aspect	160	46.02%
**Satisfaction of values**		
Creating a family	61	19.17%
Personal fulfillment	72	26.42%
Well-being of society	28	9.22%
Increasing one's skills	36	13.48%
Preparing for the future	47	13.82%
Commitment	44	12.72%
Other	40	4.70%

For the concept of decent work, we proceeded in the same way, reading and correcting the answers first and analyzing the “word frequency” to identify the nodes, excluding the “nonsignificant words.” Table 3 shows the results of this query.

**Table 3:** Word frequency for the concept of decent work.

**Word**	**Count**	**Word**	**Count**
Work	219	Rules	7
Decent	37	Rights	6
Money	37	Law	6
Like	32	Fatigue	6
Respect	26	To gain	5
Good	21	Wage	5
Correct	17	To live	5
Honest	16	Beautiful	4
All	15	Capacity	4
People	12	Important	4
Family	11	Illegal	4
Keep	7	Security	4

Based on the word frequency, we have codified the nodes as shown in Table 4. The nodes were (1) respect of rights and duties, which refers to the respect of rights and duties in the workplace. The examples that have been categorized into this node are “decent work is a job in which you are respected by your principal” (participant no. 66) or “it is a job that is not illegal” (participant no. 84). (2) Economic aspect. Similarly to the concept of work, this node refers to the perception of decent work as something useful for earning money. Examples are “it is a job that makes individuals earn” (participant no. 201) or “it is a job that guarantees money” (participant no. 306). (3) Personal well-being refers to a view of decent work as something that allows people to be happy. Examples of this coding are “decent work is when a person goes to work with pleasure and is happy with what he/she does” (participant no. 73) or “it is a way to have the opportunity to do something that gives satisfaction” (participant no. 114). (4) Good job. In this node, we have categorized the answers of those who identify decent work as a job well-done or that allows to feel important for example, “it is a job well-done” (participant no. 97) or “decent work means doing an important job” (participant no. 88). (5) Commitment. It refers to the commitment required by decent work. Examples of this coding are “decent work is that work in which you put your soul and commitment into it” (participant no. 62) or “it is a work that is carried out with commitment” (participant no. 121). (6) Equity. This node refers to the perception of equality of treatment between people of different gender or race, for example “it is a job that does not distinguish between sexes and is the same for everyone” (participant no. 281) or “it is a job that it provides for equality between men and women, between white and black people” (participant no. 289). (7) Security refers to job security. The following examples are part of this node: “it is a work that allows to always being safe, with a salary that allows you not to live in conditions of poverty” (participant no. 12) or “it is a job in healthy hygienic conditions, which does not make the worker uncomfortable” (participant no. 232). (8) Family maintenance refers to the possibility of maintaining one's family through decent work. For example, “it's a job you do to maintain the family” (participant no. 26) or “it serves to support the family” (participant no. 104). Some definitions of work were negative, for example, “it's a job that you don't like, and you don't like” (participant no. 43) or “it's the job that lousy people who love money offer you to who doesn't care about you.” We have classified these last responses in a node called “negative idea.” Finally, many participants did not answer or gave non-classifiable answers, such as “all jobs are decent” (participant no. 148) or “the jobs are all the same” (participant no. 221). We have classified these last answers, together with the non-answers, in a separate node called “other.”

**Table 4:** Nodes, references, and coverage for the concept of decent work.

**Node**	**References**	**Coverage**
Respect of rights and duties	89	27.87%
Economic aspect	45	17.57%
Personal well-being	42	14.86%
Good job	36	11.54%
Commitment	20	8.12%
Equity	15	6.40%
Security	9	5.17%
Family maintenance	6	2.15%
Negative idea	22	7.53%
Other	95	8.98%

### Phase 2

Based on the results obtained in the first phase, we calculated a complexity score of the representations of work and decent work based on the number of categories that the participants had identified.

Regarding the concept of work, we formed four groups: the first group (no idea) is formed by 40 participants (13%) and includes those who have been classified as “other,” that is, who have not expressed any idea about the concept of work. The second group (little complex) included those who identified only one category; 44.5% of students belong to this group, 137 participants who have a one-dimensional view of the work. The third group (moderately complex) included 88 students, 28.6%, who identified two categories, showing that they have a more complex representation of the work. The fourth group (complex) included 43 participants, 14%. The latter are those who have shown to have a more complex representation of the work as they have envied three or more categories. Based on these subdivisions we found significant differences between the four groups.

Before doing this, we evaluated the normality of the distribution for the evaluated dimensions using the Shapiro–Wilk test for each dimension and each group. The non-normal distribution (*p* < 0.05) of five out of six factors justified the use of nonparametric methods for data analysis. Kruskal–Wallis tests indicated significant differences for all factors. Table 5 shows the result of this analysis. There are significant differences between group “moderately complex” and group “complex” about all the dimensions considered. In particular, the group “complex” shows higher scores. In the same direction, with higher scores for the group “complex,” significant differences emerged with the group “no idea” and, “little complex” for the dimensions control, curiosity, confidence, and the total career adaptability score.

**Table 5:** Scores for the six factors and results of pairwise comparisons among groups.

**Factor**	**Group (*N*, %)**	**Median (Mean)**	**Kruskal-Wallis tests *p*-value**	**Pairwise comparisons results**			
Self-efficacy	1 (40, 13)	33.50 (32.83)	0.004	A			
	2 (137, 44.5)	34 (34.35)			B		
	3 (88, 28.6)	34 (33.43)				C	
	4 (43, 14)	36 (37.49)				C	D
Concern	1 (40, 13)	22.50 (21.20)	0.001	A			
	2 (137, 44.5)	23 (22.36)			B		
	3 (88, 28.6)	22 (21.40)				C	
	4 (43, 14)	24 (24.14)				C	D
Control	1 (40, 13)	22 (21.70)	0.000	A			
	2 (137, 44.5)	22 (22.77)			B		
	3 (88, 28.6)	23 (22.30)				C	
	4 (43, 14)	25 (24.93)		A	B	C	D
Curiosity	1 (40, 13)	21 (20.53)	0.000	A			
	2 (137, 44.5)	22 (21.43)			B		
	3 (88, 28.6)	22 (21.34)				C	
	4 (43, 14)	25 (24.21)		A	B	C	D
Confidence	1 (40, 13)	22 (21.50)	0.002	A			
	2 (137, 44.5)	22 (22.86)			B		
	3 (88, 28.6)	22 (21.75)				C	
	4 (43, 14)	26 (25.19)		A	B	C	D
Career adaptability	1 (40, 13)	84.50 (84.83)	0.000	A			
	2 (137, 44.5)	89 (89.42)			B		
	3 (88, 28.6)	86 (86.78)				C	
	4 (43, 14)	99 (98.47)		A	B	C	D

For the concept of decent work, we have formed three groups: the first group (no idea or negative idea) is formed by those who have no idea of what decent work is or who express it negatively: they are those who have been classified as “negative idea” or “other.” It is composed of 117 participants (38%). In the second group (little complex), we have included those who have a one-dimensional idea of the concept of decent work (121 participants, 39.3%). Finally, 70 students (22.7%) formed the third group (moderately complex) as they showed a more articulated idea of the concept of decent work. In any case, few students have identified more than three categories, so we decided to form only three groups and not four.

Shapiro–Wilk test revealed that the distributions were normal for the three groups only for one dimension; for this reason, we applied the Kruskal–Wallis tests with pairwise comparisons. Table 6 shows the results. Significant differences emerged in the scores of three dimensions: self-efficacy, concern, and the total career adaptability score. Specifically, the “no idea or negative idea” group has a lower score than the “moderately complex” group in terms of self-efficacy, concern, and the career adaptability total score. The same difference is found between the “little complex” group and the “moderately complex” group but only for the concern size and the career adaptability total score.

**Table 6:** Scores for the six factors and results of pairwise comparisons among groups.

**Factor**	**Group (*N*, %)**	**Median (Mean)**	**Kruskal-Wallis tests *p*-value**	**Pairwise comparisons results**		
Self-efficacy	1 (117, 38)	34 (33.68)	0.027	A		
	2 (121, 39.3)	34 (34.06)			B	
	3 (70, 22.7)	36 (35.87)		A		C
Concern	1 (117, 38)	22 (21.71)	0.006	A		
	2 (121, 39.3)	22 (21.93)			B	
	3 (70, 22.7)	24 (23.37)		A	B	C
Control	1 (117, 38)	22 (22.32)	0.119	A		
	2 (121, 39.3)	23 (22.76)			B	
	3 (70, 22.7)	23 (22.90)				C
Curiosity	1 (117, 38)	22 (21.44)	0.059	A		
	2 (121, 39.3)	22 (21.34)			B	
	3 (70, 22.7)	22.50 (22.64)				C
Confidence	1 (117, 38)	22 (22.17)	0.057	A		
	2 (121, 39.3)	22 (22.64)			B	
	3 (70, 22.7)	23 (23.64)				C
Career adaptability	1 (117, 38)	88 (87.64)	0.011	A		
	2 (121, 39.3)	88 (88.67)			B	
	3 (70, 22.7)	92.50 (93.31)		A	B	C

No differences were found in the other dimensions of career adaptability.

## Discussion

The study presented intended to explore the idea of work and decent work in a group of Italian adolescents. The results highlighted that the work, for this target, is mainly characterized by economic benefits and satisfaction of values. Most of the sample describes the work in positive terms; only a few emphasize its commitment in terms of sacrifice or effort. These characteristics are consistent with those identified by other authors (Chaves et al., 2004; Blustein, 2006; Ferrari et al., 2009).

Regarding the concept of decent work, it is interesting to note that many of the participants fail to define this concept or only use synonyms in describing it; moreover, some participants describe it in merely negative terms. The others identify the aspect related to the respect of rights and duties, the economic usefulness, and the personal advantages, but only a few introduce the aspects of equity and safety.

These data show that young Italians spontaneously develop their concept on work based almost exclusively on extrinsic aspects; moreover, when the adjective “decent” is added to the word “work,” the definitions also focus largely on respect for rights and duties, even if the concept of decent work often remains poor and difficult to define.

The results also showed that those who have a more complex concept of work also show greater conviction in the possibility of being able to reach their goals and in the ability to adapt to changes in the world of work. Similarly, those with a more complex definition of decent work also show higher levels of self-efficacy and career adaptability. In the latter case, the dimension of career adaptability most involved is the tendency to worry in a positive way about one's future. Thus, a more complex idea of work and decent work is associated with higher levels of self-efficacy and career adaptability and may be important for the individual to develop a flexible and comprehensive sense of work and decent work (Chaves et al., 2004).

## Conclusions and Limitations

Although these results contribute to enriching knowledge in the field of career education and counseling, it is necessary to underline some limitations of the study. The measures used, first, are self-reports and therefore subject to the limitations typical of these tools (social desirability, self-evaluation). Second, references should be made to the cultural influences that exist not only between different countries but also within the same country where the study was conducted, Italy. The study was conducted in southern Italy, a part of the country where job availability is reduced, and consequently, unemployment is higher than in other parts. This could probably influence the definition of aspects of work and decent work. Furthermore, the concepts of work and decent work are complex, and it might seem simplistic trying to approach this complexity in a single sentence. Furthermore, other contextual variables, such as socioeconomic background, individual and familiar experiences, and high school attended, could play a role in the construction of the concepts studied. However, we are convinced that these results may have an impact on career counseling as they reflect on the importance of helping adolescents not only to develop personal dimensions but also to better define the surrounding context. Other researchers could be stimulated to broaden the results using a more complex methodology for studying the complexity of work and decent work.

Our results, however, confirm the need to create interventions through which, since adolescence, we can help the students to reflect on the concepts of work and decent work, enlarging their possibilities to think to their future work. The knowledge of the ideas that people have about work is important as these ideas can greatly influence the way individuals make choices and decisions, characterizing their career and life (Ferrari et al., 2008). In the context of career counseling, therefore, knowing what their clients think about work can help counselors to support them in the transition phases.

The concept of decent work is related to the concept of sustainability (Magnano et al., 2019), and within the 2030 agenda is one of the objectives: “Promote sustained, inclusive, and sustainable economic growth, full and productive employment and decent work for all” (Goal 8, United Nations, 2015). This is a challenge that career counseling and career education cannot escape from. In fact, considering that the 2030 agenda poses the achievement of decent work for all among its aims, we can easily create a link to the Life Design approach, born from the need to review guidance practices, adapting them to the new context and accompanying people in professional transitions to get a truly decent work (Masdonati and Dauwalder, 2010).

Adolescents will be the future workers, and it is necessary, in our opinion, that career counseling experiences also include activities that could improve the awareness about the influences of the concepts of work and decent work on the future students' plans. However, a career education program based on the Life Design paradigm cannot disregard the role of positive dimensions such as career adaptability or self-efficacy. Both are dimensions linked to career choices and the well-being of individuals (Capri et al., 2012; Magnano et al., 2021a). Indeed, adolescents with higher levels of career adaptability have greater success in career transition phases (Patton et al., 2002; Creed et al., 2003; Germeijs and Verschueren, 2007; Neuenschwander and Garrett, 2008) and positive development (Gore et al., 2003; Skorikov, 2007; Skorikov and Vondracek, 2007). Self-efficacy has been studied in relation to many variables, such as mental health (Najafi and Foladjang, 2007), career (Abele and Spurk, 2009), or feelings of guilt (Duggleby et al., 2014). All these aspects of life can generate specific positive or negative emotions in individuals (Kidd, 2008; Perdighe et al., 2015; Gloria and Steinhardt, 2016), and self-efficacy can serve as a supporting factor for positive experiences. Levels of self-efficacy influence career choices (Betz and Hackett, 2006), job satisfaction (Saks, 1995; Higgins et al., 2008), and career success (Valcour and Ladge, 2008).

In other studies, we have shown that career education and counseling can do a lot in this direction even starting from adolescence, helping young people to have broad ideas of work and decent work, as well as promoting dimensions, such as career adaptability and self-efficacy (Zammitti et al., 2020) or hope and optimism (Santisi et al., 2021). Furthermore, the study offers a contribution to broadening the field of career education using mixed techniques (Blustein et al., 2005; Schultheiss, 2005; Whiston and Rahardja, 2005).

## Data Availability Statement

The raw data supporting the conclusions of this article will be made available by the authors, under request.

## Ethics Statement

The studies involving human participants were reviewed and approved by IERB - Internal Ethic Review Board of Psychology Research - Department of Educational Sciences. Written informed consent to participate in this study was provided by the participants' legal guardian/next of kin.

## Author Contributions

AZ contributed to conceptualization, methodology, investigation, performed the statistical analysis and wrote the first draft of the manuscript. PM and GS contributed to supervision, writing and editing. All authors contributed to manuscript revision, read and agreed to the published version of the manuscript.

## Conflict of Interest

The authors declare that the research was conducted in the absence of any commercial or financial relationships that could be construed as a potential conflict of interest.
